# Long-term survival of stage I multiple myeloma given chemotherapy just after diagnosis or at progression of the disease: a multicentre randomized study

**DOI:** 10.1054/bjoc.1999.1087

**Published:** 2000-03-06

**Authors:** A Riccardi, O Mora, C Tinelli, D Valentini, S Brugnatelli, R Spanedda, A De Paoli, L Barbarano, M Di Stasi, M Giordano, C Delfini, G Nicoletti, C Bergonzi, E Rinaldi, L Piccinini, E Ascari, for the Co-operative Group of study and Treatment of Multiple Myeloma

**Affiliations:** Medicina Interna e Oncologia Medica, Direzione Scientifica, Università and Istituto di Ricovero e Cura a Carattere Scientifico Policlinico S Matteo, Pavia, 27100; Istituto di Ematologia, Università di Ferrara, Ferrara, 44100; Divisione di Medicina II, Ospedale di Legnano, Legnano, 20025; Divisione di Ematologia, Ospedale di Niguarda, Milano, 20100; Divisione di Medicina I, Ospedale di Piacenza, Piacenza, 29100; Servizio di Oncologia, Ospedale S Anna, Como, 22100; Divisione di Ematologia, Ospedale di Pesaro, Pesaro, 61100; Semeiotica Medica, Università Cattolica di Roma, Roma, 00168; Divisione di Medicina II, Ospedale di Cremona, Cremona, 26100; Divisione di Medicina I, Ospedale di Magenta, Magenta, 20013; Istituto di Oncologia, Università di Modena, Modena, 41100, Italy

**Keywords:** multiple myeloma, stage I, early or delayed treatment, survival

## Abstract

We conducted a randomized trial to evaluate whether melphalan-prednisone (MPH-P) treatment administered just after diagnosis improves survival of stage I multiple myeloma (MM). Between January 1987 and March 1993, 145 consecutive previously untreated patients with stage I MM were randomized between treatment with MPH-P (administered for 4 days every 6 weeks) just after diagnosis and treatment only at disease progression. Survival was not influenced by MPH-P treatment either administered just after diagnosis or at disease progression (64 vs 71 months respectively). Comparing the first with the second group the odds ratio of death is 1.17 (95% confidence interval 0.57–2.42;*P* = 0.64). Disease progression occurred within a year in about 50% of patients who were initially untreated. Response rate was similar in both groups, but duration of response was shorter in patients who were treated at disease progression (48 vs 79 months, *P* = 0.044). Patients actually treated at disease progression (34/70) survived shorter than those who had neither disease progression nor treatment (56 vs > 92 months;*P* = 0.005). Starting MPH-P just after diagnosis does not improve survival and response rate in stage I MM, with respect to deferring therapy until disease progression. However, patients with stage I MM randomized to have treatment delayed and who actually progressed and were treated had shorter survival than those with stable disease and no treatment. Biologic or other disease features could identify these subgroups of patients. © 2000 Cancer Research Campaign


*British Journal of Cancer* (2000) **82**(7), 1254–1260
© 2000 Cancer Research Campaign
DOI: 10.1054/ bjoc.1999.1087, available online at http://www.idealibrary.com on IDEAL®

### Long-term survival of stage I multiple myeloma given chemotherapy just after diagnosis or at progression of the disease: a multicentre randomized study

**A Riccardi[1], O Mora[1], C Tinelli[2], D Valentini[1], S Brugnatelli[1], R Spanedda[3], A De Paoli[4], L Barbarano[5], M Di Stasi[6], M Giordano[7], C Delfini[8], G Nicoletti[9], C Bergonzi[10], E Rinaldi[11], L Piccinini[12] and E Ascari[1] for the Cooperative Group of Study and Treatment of Multiple Myeloma**

[1]Medicina Interna e Oncologia Medica and [2]Direzione Scientifica, Università and Istituto di Ricovero e Cura a Carattere Scientifico Policlinico S Matteo, 27100 Pavia; [3] Istituto di Ematologia, Università di Ferrara, 44100 Ferrara; [4]Divisione di Medicina II, Ospedale di Legnano, 20025 Legnano; [5]Divisione di Ematologia, Ospedale di Niguarda, 20100 Milano; [6]Divisione di Medicina I, Ospedale di Piacenza, 29100 Piacenza; [7]Servizio di Oncologia, Ospedale S Anna, 22100 Como; [8]Divisione di Ematologia, Ospedale di Pesaro, 61100 Pesaro [9]Semeiotica Medica, Università Cattolica di Roma, 00168 Roma; [10]Divisione di Medicina II, Ospedale di Cremona, 26100 Cremona; [11]Divisione di Medicina I, Ospedale di Magenta, 20013 Magenta; [12]Istituto di Oncologia, Università di Modena, 41100 Modena, Italy

**Summary** We conducted a randomized trial to evaluate whether melphalan-prednisone (MPH-P) treatment administered just after diagnosis improves survival of stage I multiple myeloma (MM). Between January 1987 and March 1993, 145 consecutive previously untreated patients with stage I MM were randomized between treatment with MPH-P (administered for 4 days every 6 weeks) just after diagnosis and treatment only at disease progression. Survival was not influenced by MPH-P treatment either administered just after diagnosis or at disease progression (64 vs 71 months respectively). Comparing the first with the second group the odds ratio of death is 1.17 (95% confidence interval 0.57–2.42; $P = 0.64$). Disease progression occurred within a year in about 50% of patients who were initially untreated. Response rate was similar in both groups, but duration of response was shorter in patients who were treated at disease progression (48 vs 79 months, $P = 0.044$). Patients actually treated at disease progression (34/70) survived shorter than those who had neither disease progression nor treatment (56 vs > 92 months; $P = 0.005$). Starting MPH-P just after diagnosis does not improve survival and response rate in stage I MM, with respect to deferring therapy until disease progression. However, patients with stage I MM randomized to have treatment delayed and who actually progressed and were treated had shorter survival than those with stable disease and no treatment. Biologic or other disease features could identify these subgroups of patients. © 2000 Cancer Research Campaign

**Keywords**: multiple myeloma; stage I; early or delayed treatment; survival

Patients with stage I (Durie and Salmon, 1975) multiple myeloma (MM) represent about 20% of patients with this disease (Jagannath et al, 1993; Bjorkstrand et al, 1994; Cunningham et al, 1994; Riccardi et al, 1994; Bensiger et al, 1996; Vesole et al, 1996). Most of these patients have no symptoms and diagnosis is usually due to a screening laboratory work-up revealing an increased serum monoclonal component (MC) concentration (Riccardi et al, 1991).

Physicians are still faced with the treatment options for these patients, i.e. as to whether starting chemotherapy as soon as diagnosis is made or delaying it until symptoms arise due to disease progression. In fact, although some patients have stable disease for years, a relevant number of them progress to overt symptomatic MM within 12–24 months (Dimopoulous et al, 1993; Hjorth et al, 1993; Facon et al, 1995). Two randomized studies (Hjorth et al, 1993; Riccardi et al, 1994) have suggested that delaying treatment does not influence survival, as in chronic lymphocytic leukaemia (Dighiero et al, 1998), but these studies are biased by the low number of enrolled patients and by the short follow-up.

We report the long-term survival results of 145 patients who were randomized between receiving treatment with melphalan-prednisone (MPH-P) just after diagnosis or at disease progression.

### MATERIALS AND METHODS

Between January 1987 and March 1993, 145 previously untreated stage I MM patients (Table 1) from 19 centres entered two consecutive multicenter protocols [MM87: between January 1987 and March 1990 (Riccardi et al, 1994); MM90: between April 1990 and March 1993 (unpublished)], that were aimed at giving separate randomized options for first-line and maintenance treatment to patients with stage I, II and III MM. The protocols were

*Received 22 March 1999*
*Revised 6 October 1999*
*Accepted 4 November 1999*

*Correspondence to:* A Riccardi

The following centres also participated in this study: Medicina I, Ospedale di Alessandria (Dr A Pagetto); Medicina I, Ospedale di Melegnano (Prof G Santagati); Medicina I, Ospedale di Gallarate (Dr A Ceriani); Cattedra di Ematologia, Università di Parma (Dr G Dotti); Medicina B, Ospedale di Biella (Dr M Badone); Medicina Generale, Ospedale di Somma Lombardo (Dr A Daverio); Medicina C, Ospedale di Varese (Dr N Brumana); Medicina A, Ospedale di Varese (Dr G Pinotti)

**Table 1** Main characteristics of patients with stage I multiple myeloma treated with melphalan-prednisone just after diagnosis or at progression of the disease

| | **Patients treated at diagnosis** | | **Patients treated at disease progression** | | |
|---|---|---|---|---|---|
| | ***n*** | **%** | ***n*** | **%** | ***P*** |
| Patients | 75 | 100 | 70 | 100 | NS |
| M/F | 46/29 | 61/39 | 34/36 | 49/51 | NS |
| IgG/IgA | 54/21 | 72/28 | 52/18 | 74/26 | NS |
| K/L | 45/30 | 60/40 | 41/29 | 59/41 | NS |
| β2</> 4.0 μg $dl^{-1}$ | 62/13 | 83/17 | 57/13 | 82/18 | NS |
| BMPC% ≤ 10/10–20/ ≥ 20% | 6/26/43 | 8/35/57 | 5/34/31 | 7/49/44 | NS |
| With one osteolysis[a] | 17 | 23 | 18 | 26 | NS |
| With symptoms | 9 | 12 | 8 | 11 | NS |

[a]Given radiotherapy.

approved by the Clinical Research Review Board of the Internal Medicine Department of the University of Pavia, and written informed consent was obtained from each enrolled patient.

Both protocols randomized stage I MM between receiving MPH-P just after diagnosis or at disease progression. The protocol MM90 differs from protocol MM87 only in that interferon α-2β was added to all phases of treatment in patients with stage II and III, but not with stage I disease.

### Diagnosis

Diagnosis of MM required the presence of at least two of the three following features: (1) a serum and/or urine MC; (2) a bone marrow plasma cell (BMPC) infiltration greater than 20%, as evaluated on trephine BM biopsy (Riccardi et al, 1990); (3) the presence of osteolytic lesions unexplained by other causes.

Other causes of increased marrow plasmacytosis and of monoclonal gammopathy had to be carefully excluded before a diagnosis of MM was made (Riccardi et al, 1994).

### Randomizations and treatment

Upon admission, patients were staged according with Durie and Salmon (1975). Stage I MM patients had to have all the following features: Hb > 10 g $dl^{-1}$, corrected serum calcium ≤ 12 mg $dl^{-1}$, normal bone skeletal X-ray or a single lytic lesion, serum lgG or lgA < 5 or 3 g $dl^{-1}$ respectively, and daily light chain excretion < 4 g. Randomizations for both first-line and maintenance therapy (Riccardi et al, 1994) were given by a Central Secretariat at the Medicina Interna & Medical Oncology of University of Pavia. Randomizations were attributed separately for each participating centre from a computer-generated list, just after the name and the affiliation of the patient were communicated by phone or fax.

In both MM87 and MM90 protocols, as first-line policy, stage I MM were randomized, between being treated just after diagnosis with MPH (0.21 mg $kg^{-1}$ $day^{-1}$ orally, days 1–4) and P (0.50 mg $kg^{-1}$ $day^{-1}$ orally, days 1–10), given at 6-week intervals for 6 courses or receiving the same treatment at progression of the disease.

Response was evaluated after 6 courses of MPH-P, according to slightly modified (Riccardi et al, 1994) clinical criteria adopted by the SECSG (Cohen et al, 1979).

Criteria were as follows: (a) reduction in MC; (b) decrease in BMPC of at least 20% or return to less than 20%, as evaluated on BM imprints before and after treatment; (c) a 2 g $dl^{-1}$ rise in Hb concentration in anaemic patients (Hb < 11 g $dl^{-1}$) sustained for more than 4 weeks; (d) return of serum calcium and blood urea nitrogen (BUN) to normal values; (e) elevation of serum albumin up to or greater than 3 g $dl^{-1}$ in the absence of other causes of hypoalbuminaemia; (f) absence of progression of skeletal lytic lesions.

Complete response (CR) was a > 50% reduction in MC and a response in more than half of the other parameters. Partial response (PR) was a 25–50% reduction in MC and a response in more than half of the other parameters. No response (NR) was the no fulfillment of the above criteria for CR and PR. Progression was a > 25% increase in MC and/or an increase in BMPC of at least 20% and/or worsening of laboratory parameters (mainly haemoglobin, serum calcium and BUN) and/or of skeletal lytic lesions.

Patients who had CR or PR were randomized between receiving additional courses of MPH-P until maximum reduction in MC (i.e. the plateau phase) was achieved (Riccardi et al, 1994) and then stopping all cytostatics until relapse, or continuing therapy indefinitely until relapse, as a maintenance.

Patients who had response to MPH-P and then relapsed while on no maintenance were retreated with MPH-P until second relapse.

Patients who were resistant to MPH-P, those who had stable disease after MPH-P and then progressed, those who relapsed while on MPH-P maintenance, and those who had second relapse after second MPH-P treatment were treated, as second-line treatment, with the association of peptichemio (PTC; Istituto Sieroterapico Milanese, Milan, 0.8 mg $kg^{-1}$ $day^{-1}$ by intravenous (i.v.) infusion, days 1, 3 and 5), vincristine (VCR; 0.025 mg $kg^{-1}$ $day^{-1}$, maximal dose 2 mg, days 1 and 14) and P (0.4 mg $kg^{-1}$ $day^{-1}$, days 1–7) given every 28 days for 4 courses. Patients who achieved response with this second-line treatment continued to be treated with this schedule until relapse (Figure 3).

### Follow-up

It has been detailed elsewhere (Riccardi et al, 1994). Briefly, performance status, blood and 24-h urine laboratory parameters, BM examination and skeletal X-rays were assessed at diagnosis and repeated every 2–3 months throughout the induction period. Then these examinations were repeated every 3–6 months or at longer intervals, as needed from clinical indications.

*© 2000 Cancer Research Campaign*

**Table 2** Additional laboratory characteristics of patients with stage I multiple myeloma treated with melphalan-prednisone just after diagnosis or at progression of the disease

| Parameter | Patients treated at diagnosis | | Patients treated at disease progression | | |
|---|---|---|---|---|---|
| | Median | Range | Median | Range | *P* |
| ESR (mm 1st h) | 47.0 | 4–124 | 40.0 | 1–130 | NS |
| Hb (g dl$^{-1}$) | 12.8 | 9.3–15.6 | 13.3 | 10.7–17.3 | NS |
| WBC (×10$^{9}$ l$^{-1}$) | 6.1 | 2.8–20.6 | 6.3 | 3.3–26 | NS |
| PLT (×10$^{9}$ l$^{-1}$) | 227.0 | 118–479 | 231.0 | 100–429 | NS |
| Creatinine (mg dl$^{-1}$) | 1.0 | 0.6–2.1 | 0.9 | 0.6–1.9 | NS |
| Serum albumin (g dl$^{-1}$) | 4.1 | 2.6–5.3 | 4.2 | 2.1–5.2 | NS |
| Serum MC (g dl$^{-1}$) | 2.2 | 0.5–4.6 | 1.9 | 0.5–3.3 | NS |
| Normal Ig (%) | 0.52 | 0.08–2.8 | 0.58 | 0.02–1.7 | NS |
| Alkaline phosphatase (U dl$^{-1}$) | 137.0 | 24–302 | 134.0 | 21–317 | NS |
| Uric acid (mg dl$^{-1}$) | 5.2 | 2.8–10 | 5.0 | 1.6–8.7 | NS |
| S-Ca$^{2+}$ (mg dl$^{-1}$) | 9.2 | 8.0–11.8 | 9.4 | 8.4–10.9 | NS |
| U-Ca$^{2+}$ (mg 24 h$^{-1}$) | 8.5 | 0.08–42.4 | 11.4 | 0.04–227 | NS |
| BJ proteinuria (g 24 h$^{-1}$) | 1.28 | 0.1–11 | 0.9 | 0.1–10 | NS |

**Table 3** Stage I MM patients: response to melphalan-prednisone (MPH-P) according with randomization

| | Patients treated at diagnosis | Patients treated at disease progression | *P* |
|---|---|---|---|
| Evaluable/entered patients | 75/75 | 34/34 | – |
| Overall response, *n* (%) | 30 (40) | 19 (55) | NS |
| Complete response, *n* (%) | 8 (11) | 5 (15) | NS |
| Partial response, *n* (%) | 22 (29) | 14 (41) | NS |
| Stable disease, *n* (%) | 40 (53) | 12 (35) | NS |
| Progressive disease, *n* (%) | 5 (7) | 3 (9) | NS |
| Median duration of response[b], months | 79 | 48 | 0.044 |

[a]Evaluable/entered patients = patients who were evaluable for response over those who received MPH-P; [b]patients who had complete or partial response after MPH-P treatment.

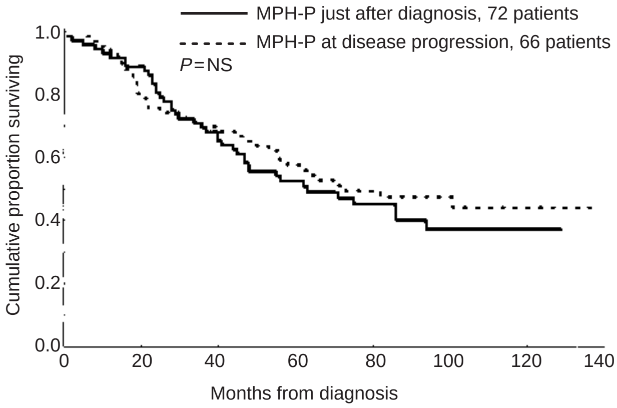

**Figure 1** Survival of patients with stage I multiple myeloma according to time of starting melphalan-prednisone (MPH-P) therapy

### Data collection

Information on the occurrence and on the duration of response were obtained from the brochure records. Duration of response is calculated from the end of successful induction therapy until relapse, and censored were surviving patients who did not have a relapse during the follow-up (patients who died before relapse were considered as events). Survival is the time from randomization to death, as obtained from the brochure records or death certificate based search. Causes of death were divided into those related and those unambiguosly unrelated to MM.

### Statistical analysis

The statistical analysis was carried out on an intention-to-treat basis. Features that could be prognostic for survival were searched by both the univariate analysis and the Cox multivariate regression analysis of the clinical, laboratory and radiologic parameters listed in Tables 1 and 2. The Cox analysis also included whether the patient started the treatment or not.

Differences in the response rate among the different groups of patients were tested by the contingency table $\mu\chi^2$ test. Survival analysis was based on Kaplan–Meier estimates, the log-rank test and the Cox regression model. All *P* are two-sided and adjusted for repeated analysis.

### RESULTS

The results of this study are reported in Tables 1–6 and Figures 1 and 2. Overall, 145 patients entered the study. Seventy-five of them (median age = 69 years, range 39–88) were treated just after diagnosis and 70 (median age = 68 years, range 33–85) at disease progression. The slight imbalance between the two arms is due to the fact that randomization was separately done for each centre and that two patients randomized to have therapy delayed were later found to be stage II MM. All patients who were randomized to immediate or delayed treatment actually followed the assigned randomization. The main clinical and laboratory characteristics of the two groups were similar and are detailed in Tables 1 and 2.

Eighty-eight per cent of patients had no disease-related symptoms, and the diagnosis was from the chance finding of a serum MC on routine haematochemical tests. Nine per cent of patients complained of bone pain at the site of the osteolyse, most often located in the spine, chest and pelvis. Patients with symptomatic or asymptomatic osteolysis were given radiotherapy (Table 1). Three

 *© 2000 Cancer Research Campaign*

**Table 4** Patients with stage I multiple myeloma who were randomized to be treated with melphalan-prednisone (MPH-P) at disease progression: main characteristics of patients who had and did not have disease progression and treatment

| | Patients with disease progression and treatment | | Patients without disease progression and treatment | | |
|---|---|---|---|---|---|
| | *n* | % | *n* | % | *P* |
| Patients | 34 | 100 | 36 | 100 | NS |
| M/F | 16/18 | 47/53 | 18/18 | 50/50 | NS |
| IgG/IgA | 24/10 | 71/29 | 28/8 | 78/22 | NS |
| K/L | 21/13 | 62/38 | 20/16 | 56/44 | NS |
| β2 </> 4.0 μg dl$^{-1}$ | 26/8 | 76/24 | 32/4 | 89/11 | NS |
| With one osteolysis | 8 | 24 | 10 | 28 | NS |
| BMPC% ≤ 10/10–20/≥20% | 2/15/17 | 7/44/49 | 3/19/14 | 7/54/39 | NS |
| With symptoms | 4 | 12 | 5 | 15 | NS |

**Table 5** Additional laboratory characteristics of patients with stage I multiple myeloma treated with melphalan-prednisone at disease progression and of patients who did not have disease progression and treatment

| Parameter | Patients with disease progression and treatment | | Patients with neither disease progression nor treatment | | |
|---|---|---|---|---|---|
| | Median | Range | Median | Range | *P* |
| ESR (mm 1st h) | 56.0 | 5–120 | 33.0 | 1–130 | NS |
| Hb (g dl$^{-1}$) | 13.3 | 10.7–17 | 13.7 | 10.9–17.3 | NS |
| WBC (×10$^9$ l$^{-1}$) | 5.7 | 3.3–10.7 | 6.8 | 3.6–26 | 0.01 |
| PLT (×10$^9$ l$^{-1}$) | 221.0 | 154–429 | 243.0 | 100–415 | NS |
| Creatinine (mg dl$^{-1}$) | 0.9 | 0.7–1.9 | 0.9 | 0.6–1.3 | NS |
| Serum albumin (g dl$^{-1}$) | 4.3 | 3.4–5.2 | 4.1 | 2.1–5.2 | NS |
| Serum MC (g dl$^{-1}$) | 2.1 | 0.6–3.3 | 1.9 | 0.5–3.2 | 0.04 |
| Normal Ig (%) | 0.48 | 0.1–1.4 | 0.59 | 0.02–1.7 | NS |
| Alkaline phosphatase (U dl$^{-1}$) | 135.0 | 21–317 | 134.0 | 70–286 | NS |
| Uric acid (mg dl$^{-1}$) | 5.0 | 1.6–8.7 | 5.2 | 3.0–8.7 | NS |
| S-Ca$^{2+}$ (mg dl$^{-1}$) | 9.5 | 8.6–10.4 | 9.4 | 8.4–10.9 | NS |
| U-Ca$^{2+}$ (mg 24 h$^{-1}$) | 11.4 | 0.14–148 | 12.5 | 0.04–227 | NS |
| BJ proteinuria (g 24 h$^{-1}$) | 0.5 | 0.25–10 | 0.9 | 0.1–10 | NS |

per cent of patients complained of modest weakness and/or fatigue during the last 6 months.

At the time of this analysis (March 1998), 138 patients have completed follow-up and are evaluable for both response and survival. Seven patients (three randomized to being treated just after diagnosis and four at disease progression) have been lost to follow-up after a period of 45 months (range 18–72) and were evaluable only for first response.

Of the 138 fully evaluable patients, 77 (56%) have died. The median follow-up of all patients is 65 months and that of living patients is 93 months (range 60–139).

### Control of the disease

In the 75 patients treated with MPH-P just after diagnosis, the overall response rate was 40% and the median duration of first response was 79 months (Table 3). No patient fulfilled the criteria for response without receiving treatment.

Thirty-four (48%) of the 70 patients who were randomized to have delayed treatment needed MPH-P because of disease progression. The median time to progression was 13 months (range 3–91). Treatment was required within 12 months in 16 out of 34 patients, between 13 and 36 months in 12 patients, and later in six patients.

Causes of disease progression and of starting treatment was a sustained, although asyptomatic, increase in MC in 12 patients, the appearance of a new and/or the enlargement of a preexisting bone lesion in 11 patients (without MC increase in two patients), the occurrence of anaemia (Hb < 10 g dl$^{-1}$) in nine patients (without MC increase in three patients), hypercalcaemia in one patient and renal failure in one patient (both with MC increase). In the three patients with disease progression diagnosed as due to isolated anaemia, the BMPC% had risen by 12–18%. Following progression, 19 patients still had stage I disease, while 12 entered stage II and three stage III disease. Two patients with worsening bone disease had vertebral compression fractures and received either chemo- and radiotherapy.

Overall response rate was 55% and the median duration of first response was 48 months ($P = 0.044$ with respect to response duration in patients treated just after diagnosis) (Table 3).

Among the 49 patients who responded to MPH-P a second response to this therapy was seen in eight (39%) of the 25 patients randomized to stop treatment until relapse.

A response to PTC-VCR-P was seen in 27% of the 72 evaluable patients who either progressed while on first MPH-P therapy

© 2000 Cancer Research Campaign

**Table 6** Causes of death of patients with multiple myeloma (MM) who were randomized to being treated just after diagnosis or at progression of the disease

| | Patients treated at diagnosis | Patients treated at disease progression |
|---|---|---|
| Patients who died, no | 41 | 35 |
| Patients whose cause of death is known, *n* | 25 | 23 |
| Causes related to MM, *n* (%) | 19 (76) | 16 (70) |
| Infections, *n* | 7 | 8 |
| Renal insufficiency, *n* | 5 | 4 |
| Hypercalcaemia, *n* | 4 | 2 |
| Other, *n* | 3 | 2 |
| Causes unrelated to MM, *n* (%) | 6 (24) | 7 (30) |
| Stroke, *n* | 3 | 2 |
| Myocardial infarction, *n* | 0 | 2 |
| Heart failure, *n* | 1 | 0 |
| Acute leukaemia and tumours, *n* | 2 | 1 |

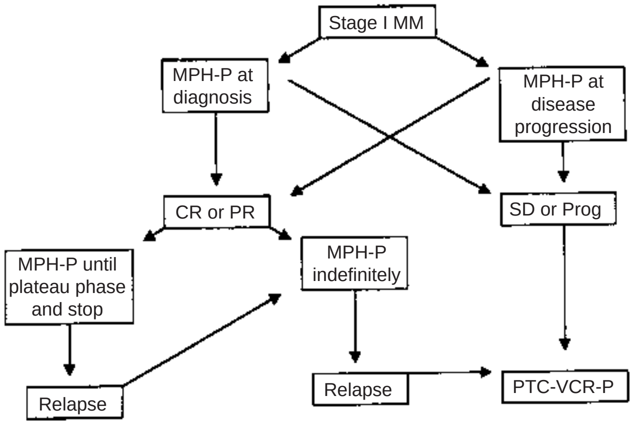

**Figure 3** Flow diagram of treatment of stage I multiple myeloma (MPH-P = melphalan-prednisone; PTC-VCR-P = peptichemio-vincristine-prednisone; CR = complete response; PR = partial response; SD = stable disease; Prog = progression)

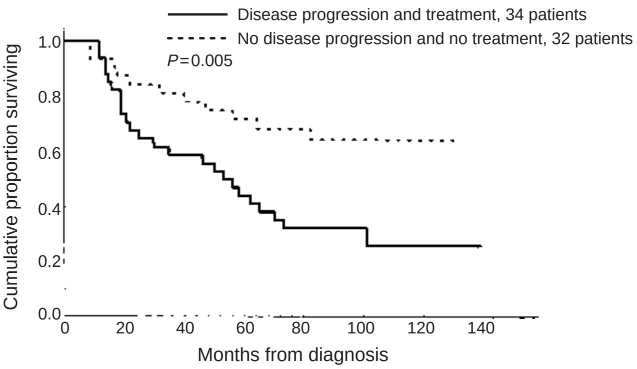

**Figure 2** Survival of patients with stage I multiple myeloma randomized to be treated at disease progression according with the need of treatment

(five patients), had stable disease after MPH-P and then progressed (38 patients), relapsed while on MPH-P maintenance (23 patients), or had second relapse after second MPH-P treatment (six patients).

### Survival duration

For all stage I MM patients, median survival was 69 months and not influenced by the type of initial randomization, i.e. starting MPH-P just after diagnosis (64 months) or at progression of the disease (71 months) (Figure 1). Response duration was independent on response to MPH-P. Comparing the first with the second group the odds ratio of death is 1.17 (95% confidence interval 0.57–2.42; $P = 0.64$).

Median survival was similar in 35 patients with (63 months) and 103 without osteolyses (59 months). Median survival was 47 and 81 months ($P$ = ns) for the 17 patients with osteolyses treated just after diagnosis and for the 18 patients with osteolyses treated at disease progression respectively. The two patients who had vertebral compression while untreated survived 68 and 71 months.

No prognostic feature (Tables 1 and 2) for survival was found by both the univariate and Cox regression analysis.

Among patients randomized to have treatment delayed, those who had disease progression and were treated fared worse (median survival = 56 months) than those who had no disease progression and are still untreated (median survival > 92 months) ($P = 0.005$) (Figure 2). Median age was similar in these two sub-groups (69 (range 39–88) and 67 (range 38–78) years respectively) and no major clinical and/or laboratory difference was found between them, except that patients who had disease progression tended to have lower WBC count and higher MC levels (Tables 4 and 5). The Cox multivariate analysis failed to add further information.

### Causes of death

Causes of death were assessable with certainty in 48 of the 76 patients who died. There were no differences in causes of death between patients randomized to being treated just after diagnosis or at progression of the disease (Table 6).

### DISCUSSION

The analysis of stage I MM patients included into the protocols MM87 and MM90 indicates that deferring treatment is a reasonable alternative to immediate chemotherapy. In fact, the presented long-term survival data (the median follow-up for living patients is 93 months) indicate that starting MPH-P just after diagnosis does not prolong long-term survival, with respect to starting treatment at disease progression.

These data confirm those from two other randomized studies (Hjorth et al, 1993; Riccardi et al, 1994) that, however, included a more limited number of cases (50 and 74 respectively) with a shorter follow-up (the median follow-up for living patients was 48 and 51 months respectively). With respect to the study of Hjorth et al (1993), where patients with lytic bone disease were excluded, some of our stage I MM had an osteolyse, but this did not influence survival, irrespective of time of starting therapy. Also, causes of death were similar and unrelated to time of therapy.

The advantage of delaying treatment in all stage I MM is of avoiding the MPH-induced myelosuppression and the necessity of closing follow-up in patients for whom there is no curative therapy. Theoretically, bone marrow transplantation (BMT) may cure them, but a number of factors limits its use. On one hand, young age and a good initial response to conventional

 © 2000 Cancer Research Campaign chemotherapy are prerequisites for BMT (Bataille and Harousseau, 1997; Riccardi et al, 1998). On the other hand, several series (Fermand et al, 1993; Harousseau et al, 1995; Attal et al, 1996; Marit et al, 1996) exclude just stage I MM from BMT, due to their intrinsic good prognosis.

Delaying MPH-P could also reduce an increased occurrence of second tumours possibly linked to the longlasting alkylating therapy. This has been reported by Bergsagel (1988), although not confirmed in this study, as well as in chronic lymphocytic leukaemia patients treated with chlorambucil (Dighiero et al, 1998).

From Hjorth et al's (1993) study and the present series, a possible disadvantage of deferring therapy is that about 2–3% of untreated patients experience vertebral compression. Although this fact does not influence survival, untreated patients must be well informed and promptly examined whenever clinical and/or laboratory data suggest disease progression.

A more general point is that the practical importance of distinguishing the monoclonal gammopathies of unknown significance (MGUS) from early MM is diminished, because treatment is to be delayed until progression in both diseases. This is true despite the fact that the two entities still maintain laboratory and, especially, clinical differences. As a matter of fact, the high cut-off point of 20% BMPC we used to separate MGUS from stage I MM in patients without bone disease discriminated two populations of patients having different clinical disease courses. In fact, the rate of malignant transformation and progression of MGUS to MM is low and continuous over time. In our series, the 10% of 735 MGUS have experienced progression to MM at a median follow-up of 5.8 years (unpublished data). The 30% of patients experience it after 15 years from diagnosis (Kyle, 1993). On the contrary, the progression of stage I MM occurred within 1–2 years in the 26–56% of patients (Dimopoulos et al, 1993; Hjorth et al, 1993; Facon et al, 1995; present series). This prompts a closer follow-up for stage I MM than for MGUS.

A question is that patients with stage I MM randomized to have treatment delayed and who actually progressed and were treated fared worse than those who had no disease progression and then were untreated, despite no major clinical or laboratory differences between the two groups were apparent. Certainly, patients who require treatment due to disease progression have or acquire increased biologic aggressiveness of a number of plasma cell clones (for example, marked by aneuploidy) (Montecucco et al, 1984) and a number of them enter stage II and III disease. Against these clones, MPH-P treatment is overall less effective, as indicated by the shorter duration of response in the overall group of stage I, II and III patients treated at progression than in stage I patients treated just after diagnosis.

Then differences between plasma cell clones exist and have to be searched, including, beside aneuploidy (Montecucco et al, 1984), high proliferative activity (Drewinko et al, 1981; Riccardi et al, 1985), plasmablastic cytology (Riccardi et al, 1990), c-*myc*, N-*ras* and K-*ras* oncogene mutations (Danova et al, 1990), and CD19, CD28, CD36, LFA-1 and VLA-5 expression (Bataille and Harousseau, 1997). An abnormal magnetic resonance imaging in the bone marrow could also increase the risk of progression (Van de Berg et al, 1996; Mariette et al, 1999).

As a conclusion, deferring therapy until disease progression does not compromise survival duration in stage I MM. Hence, treatment may be delayed at time of disease progression even if patients must be well informed about risk, i.e. vertebral compression can occur while deferring therapy. Because patients who have treatment delayed and are treated at disease progression fare worse than those who had no disease progression and are still untreated, biologic or other disease features have to be searched for to identify these subgroups of stage I MM patients.

### ACKNOWLEDGEMENTS

This research was supported by AIRC (Associazione Italiana per la Ricerca sul Cancro, Milano), by CNR (Consiglio Nazionale delle Ricerche, Roma, PF ACRO, grant no. 96.00626.PF39), by IRCCS Policlinico San Matteo (Pavia), and by MURST (Ministero dell'Università e della Ricerca Scientifica e Tecnologica, Roma).

### REFERENCES

Attal M, Harousseau JL, Stoppa AM, Sotto JJ, Fuzibet JG, Rossi JF, Casassus P, Maisonneuve H, Facon T, Ifrah N, Payen C and Bataille R for the Intergroup Francaise du Myelome (1996) A prospective, randomized trial of autologous bone marrow transplantation and chemotherapy in multiple myeloma. *N Engl J Med* **335**: 91–97

Bataille R and Harousseau JL (1997) Multiple myeloma. *N Engl J Med* **336**: 1657–1664

Bergsagel DE (1988) Chemotherapy of myeloma: drug combinations versus single agents, and overview, and comments on acute leukemia in myeloma. *Hematol Oncol* **6**: 159–166

Besinger WI, Rowley SD, Demirer T, Lilleby K, Schiffman K, Clift RA, Appelbaum FR, Fefer A, Barnett T, Storb R, Chauncey T, Maziarz RT, Klarnet J, McSweeney P, Holmberg L, Maloney DG, Weaver CH and Buckner CD (1996) High dose therapy followed by autologous hematopoietic stem-cell infusion for patients with multiple myeloma. *J Clin Oncol* **14**: 1447–1456

Björkstrand B, Goldstone AH, Ljungman P, Brandt L, Brunet S, Carlson K, Prentice G, Cavo M, Samson D, De Laurenzi A, Verdonck LF, Proctor S, Ferrant A, Sierra J, Auzanneau G, Troussard X, Gravett P, Remes K and Gahrton G for the European Group for Bone Marrow Transplantation (1994) Prognostic factors in autologous stem cell transplantation for multiple myeloma: an EBMT registry study. *Leuk Lymphoma* **15**: 265–272

Cohen HJ, Silberman HR, Larsen WE, Johnson L, Bartolucci and Durant JR (1979) Combination chemotherapy with intermittent 1-3(bis-2-chloroethyl)-1-nitrosurea (BCNU), cyclophosphamide and prednisone for multiple myeloma. *Blood* **54**: 824–836

Cunningham D, Paz-Arez L, Milan S, Powles R, Nicolson M, Hickish T, Selby P, Treleavan J, Viner C, Malpas J, Slevin M, Findlay M, Raymond J and Gore ME (1994) High dose melphalan and autologous bone marrow transplantation as consolidation in previously untreated myeloma. *J Clin Oncol* **12**: 759–763

Danova M, Riccardi A, Ucci G, Luoni R, Giordano M and Mazzini G (1990) Ras oncogene expression and DNA content in plasma cell dyscrasia: a flow cytofluorimetric study. *Br J Cancer* **62**: 781–785

Dighiero G, Maloum K, Desablens B, Cazin B, Navarro M, Leblay R, Leporrier M, Jaubert J, Lepeu G, Dreyfus B, Binet JL and Travade P for the French Cooperative Group on Chronic Lymphocytic Leukemia (1998) Chlorambucil in indolent chronic lymphocytic leukemia. *N Engl J Med* **338**: 1506–1514

Dimopoulos MA, Moulopoulos A, Smith T, Delasalle KB and Alexanian R (1993) Risk of disease progression in asymptomatic multiple myeloma. *Am J Med* **94**: 57–61

Drewinko B, Alexanian R, Boyer H, Barlogie B and Rubinow SI (1981) The growth rate of human myeloma cells. *Blood* **57**: 333–338

Durie BGM and Salmon SE (1975) A clinical staging system for multiple myeloma. *Cancer* **36**: 842–854

Facon T, Menard JF, Michaux JL, Euller-Ziegler L, Bernard JF, Grosbois B, Daragon A, Azais I, Courouble Y, Kaplan G, Laport JP, De Gramont A, Duclos B, Leonard A, Mineur P, Delannoy A, Jouet JP, Bauters F and Monconduit M (1995) Prognostic factors in low tumour mass asymptomatic multiple myeloma: a report on 91 patients. *Am J Hematol* **48**: 71–75

Fermand JP, Chevret S, Ravaud P, Divine M, Leblonde V, Dreyfus F, Mariette X and Brouet JC (1993) High-dose chemoradiotherapy and autologous blood stem cell transplantation in multiple myeloma: results of a phase II trial involving 63 patients. *Blood* **82**: 2005–2009.

© 2000 Cancer Research Campaign

Harousseau JL, Attal M, Divine M, Marit G, Leblond V, Stoppa AM, Bourhis JH, Caillot D, Boasson M, Abgrall JF, Facon T, Linassier C, Cahn JY, Lamy T, Troussrd X, Gratecos N, Pignon B, Auzanneau G and Bataille R (1995) Autologous stem cells transplantation after first remission induction treatment in multiple myeloma: a report of the French Registry on autologous transplantation in multiple myeloma. *Blood* **85**: 3077–3085

Hjorth M, Hellquist L, Holmberg E, Magnusson B, Rödier S and Westin J (1993) Initial versus deferred melphalan-prednisone therapy for asymptomatic multiple myeloma stage I: a randomized study. *Eur J Haematol* **50**: 95–102

Jagannath S, Barlogie B, Vesole D, Alexanian R, Tricot G and Crowley J (1993) Two-hundred sixty autotransplants (TX) for multiple myeloma (MM): prognostic factor analysis. *Blood* **82**: 198a (abstract 779).

Kyle RA (1993). "Benign" monoclonal gammopathy: after 20 to 35 years of follow-up. *Mayo Clinic Proc* **68**: 26–36

Mariette X, Zagdanski AM, Guermazi A, Bergot C, Arnould A, Frija J, Brouet JC and Fermand JP (1999) Prognostic value of vertebral lesions detected by magnetic resonance imaging in patients with stage I multiple myeloma. *Br J Haematol* **104**: 723–729

Marit G, Faberes C, Pico JL, Boiron JM, Bourhis JH, Brault P, Bernard P, Foures C, Cony-Makhoul P, Puntous M, Vezon G, Broustet A, Girault D and Reiffers J (1996) Autologous peripheral-blood progenitor-cell support following high-dose chemotherapy or chemoradiotherapy in patients with high-risk multiple myeloma. *J Clin Oncol* **14**: 1306–1313

Montecucco CM, Riccardi A, Merlini GP, Mazzini G, Giordano P, Danova M and Ascari E (1984) Plasma cell DNA content in multiple myeloma and related paraproteinemic disorders. Relationship with clinical and cytokinetic features. *Eur J Cancer* **20**: 81–89

Riccardi A, Montecucco CM, Danova M, Ucci G, Merlini GP and Ascari E (1985) Rate of M-component changes and plasma cell labeling index in 25 patients with multiple myeloma treated with Peptichemio. *Cancer Treat Rep* **69**: 971–975

Riccardi A, Ucci G, Luoni R, Castello A, Coci A, Magrini U and Ascari E for the Cooperative Group of Study and Treatment of Multiple Myeloma (1990) Bone marrow biopsy in monoclonal gammopathies: correlations between pathologic findings and clinical data. *J Clin Pathol* **43**: 469–475

Riccardi A, Gobbi P, Ucci G, Bertoloni D, Luoni R, Rutigliano L and Ascari E (1991) Changing clinical presentation of multiple myeloma. *Eur J Cancer* **27**: 1401–1405

Riccardi A, Ucci G, Luoni R, Brugnatelli S, Mora O, Spanedda R, De Paoli A, Barbarano L, Di Stasi M, Alberio F, Delfini C, Nicoletti G, Morandi S, Rinaldi E, Piccinini L, De Pasquale A and Ascari E (1994) Treatment of multiple myeloma according to extension of disease: a prospective, randomized study comparing a less with a more aggressive cytostatic policy. *Br J Cancer* **70**: 1203–1210

Riccardi L, Mora O, Brugnatelli S, Ucci G, Spanedda R, De Paoli A, Barbarano L, Di Stasi M, Giordano M, Delfini C, Nicoletti G, Morandi S, Rinaldi E, Piccinini L and Ascari E (1998) Relevance of age on survival of 341 patients with multiple myeloma treated with conventional chemotherapy: results of the MM87 prospective randomised protocol. *Br J Cancer* **77**: 485–491

Van de Berg BC, Lecouvet FE, Michaux L, Labaisse M, Malghem J, Jamart J, Maldague BE, Ferrant A and Michaux JL (1996). Stage I multiple myeloma: value of MR imaging of the bone marrow in the determination of prognosis. *Radiology* **20**: 243–246

Vesole DH, Tricot G, Jagannath S, Desikan KR, Siegel D, Dwayne B, Miller L, Cheson B, Crowley J and Barlogie B (1996) Autotransplants in multiple myeloma: what have we learned? *Blood* **88**: 838–847

 *© 2000 Cancer Research Campaign*
